# Prevalence of Cigarette Smoking Among Adult Emergency Department Patients in Canada

**DOI:** 10.5811/westjem.2020.9.47731

**Published:** 2020-11-01

**Authors:** Andrew D. Tolmie, Rebecca Erker, Taofiq Oyedokun, Emily Sullivan, Thomas Graham, James Stempien

**Affiliations:** * University of Saskatchewan, College of Medicine, Saskatchewan, Canada; † Saskatchewan Health Authority, Department of Emergency Medicine, Saskatchewan, Canada; ‡ University of Saskatchewan, Department of Emergency Medicine, Saskatchewan, Canada; § University of Saskatchewan, Department of Academic Family Medicine, Saskatchewan, Canada

## Abstract

**Introduction:**

Tobacco smoking is a priority public health concern, and a leading cause of death and disability globally. While the daily smoking prevalence in Canada is approximately 9.7%, the proportion of smokers among emergency department (ED) patients has been found to be significantly higher. The purpose of this survey study was to determine the smoking prevalence of adult ED patients presenting to three urban Canadian hospitals, and to determine whether there was an increased prevalence compared to the general public.

**Methods:**

A verbal questionnaire was administered to adult patients aged 18 years and older presenting to Royal University Hospital, St. Paul’s Hospital, and Saskatoon City Hospital in Saskatoon, Saskatchewan. We compared patients’ smoking habits to Fagerström tobacco dependence scores, readiness to quit smoking, chief complaints, Canadian Triage Acuity Scale scores, and willingness to partake in ED-specific cessation interventions.

**Results:**

A total of 1190 eligible patients were approached, and 1078 completed the questionnaire. Adult Saskatoon ED patients demonstrated a cigarette smoking prevalence of 19.6%, which is significantly higher than the adult Saskatchewan public at 14.65% (P<0.0001). Out of the smoking cohort, 51.4% indicated they wanted to quit smoking and would partake in ED-specific cessation counselling, if available. Of the proposed interventions, ED cessation counselling was most popular among patients (62.4%), followed by receiving a pamphlet (56.2%), and referral to a smokers’ quit line (49.5%).

**Conclusion:**

The higher smoking prevalence demonstrated among ED patients highlights the need for a targeted intervention program that is feasible for the fast-paced ED environment. Training ED staff to conduct brief cessation counselling and referral to community supports for follow-up could provide an initial point of contact for smokers not otherwise receiving cessation assistance.

## INTRODUCTION

Cigarette smoking is a global health epidemic which significantly influences the rates of cardiovascular, respiratory, and malignant chronic diseases. The deleterious influence of smoking on these diseases has resulted in cigarette smoking becoming a leading public health concern.^1^ Despite persistent public health campaigns addressing smoking cessation over the past few decades, the daily cigarette smoking prevalence in the Canadian population was estimated at 9.7% in 2018.^1^ In Saskatchewan, this rate was estimated to be higher, at 14.65%.^2^,^3^ Saskatchewan has a high proportion of rural-based citizens, and one of the lowest provincial population densities in Canada. This could impact the efficacy of smoking cessation initiatives, and partially explain why the provincial smoking rate is higher. Furthermore, Saskatchewan has a high proportion of indigenous people, who have a higher smoking prevalence than non-indigenous Canadians.^4^

American, Australian, and New Zealand studies have demonstrated a high proportional cigarette smoking prevalence in emergency department (ED) patients.^5^–^10^ Additionally, four of these studies compared the calculated ED smoking prevalence with the public rate, and all demonstrated an increased smoking prevalence among ED patients.^5^–^8^ However, the prevalence of cigarette smoking as well as the associated demographics and characteristics of ED patients has not been studied to a similar extent in Canada. In 2011, a study in northern Ontario found an ED prevalence nearly double the public rate, but patient smoking habits were not further explored.^11^ One other Canadian study demonstrated a smoking prevalence of 46%; however, the data was not compared to the public rate.^12^ Due to differences in population demographics, government tobacco policy, and healthcare systems, international studies may not be generalizable to a Canadian setting. As a result, additional Canadian studies are needed to address this knowledge gap.

Traditionally, smoking cessation counselling falls within the primary care provider’s scope of practice and is not usually performed in the ED. The Canadian Action Network for the Advancement, Dissemination and Adoption of Practice-informed Tobacco Treatment (CAN-ADAPTT) guidelines, an evidence-based protocol designed by the Centre for Addiction and Mental Health, recommend that all Canadian hospitals should have a system in place to help patients quit smoking.^13^ However, these programs are generally introduced at an inpatient level, which precludes their impact among ED patients who are discharged home after treatment. Furthermore, American literature has demonstrated that ED patients who smoke are less likely than non-smokers to have a primary care provider, meaning they may not be receiving cessation support elsewhere.^9^ These issues identify the need for ED-specific cessation interventions in departments with an increased smoking prevalence.

The primary objective of this survey study was to determine the smoking prevalence of adult ED patients presenting to three urban Canadian hospitals, and compare this to the prevalence of the general public. Secondary objectives included identifying trends in the actively smoking cohort through demographics, smoking habits, readiness to quit, chief complaint and Canadian Triage Acuity Scale (CTAS) score. We also assessed participant receptiveness to a variety of potential ED smoking-cessation interventions that have been trialed in other studies. These include brief motivational interviewing, distributing cessation materials, providing referrals to a smoker’s quit line, or a combination thereof.^14^–^19^ In summary, our goal was to identify whether the prevalence of ED patients who smoke is elevated in a Canadian setting, similar to previous international data. Additionally, we sought to assess whether an ED-specific cessation intervention would be beneficial in these urban hospitals and determine which cessation modalities would be best received by the smoking cohort.

**Population Health Research Capsule**

What do we already know about this issue?

*Previous international research has shown adult emergency department patients have a higher prevalence of cigarette smoking compared to respective community rates*.

What was the research question?

Is this data generalizable to three Canadian hospitals, and are patients open to ED based cessation interventions?

What was the major finding of the study?

*The ED smoking rate is higher than the provincial. 51% of patients who smoke are open to ED cessation support*.

How does this improve population health?

*This highlights the ED as a novel location for providing smoking cessation interventions to a population who may not receive cessation support elsewhere*.

## METHODS

A cross-sectional questionnaire was administered to patients at all EDs within Saskatoon, Saskatchewan. In total, the three tertiary EDs (Royal University Hospital, St. Paul’s Hospital, and Saskatoon City Hospital) accommodate 130,000 annual patient visits from Saskatoon and the surrounding area; these hospitals provide tertiary care for the entire northern half of the province. Participants were eligible if they were 18 years of age or older and able to independently answer the verbal questionnaire administered by researchers. Patients were excluded if they were confused, actively being tended to by a healthcare professional, medically unstable, unable to verbally communicate due a medical condition, or if they were unable to communicate in English.

We employed verbal administration of the questionnaire to improve accessibility for patients with literacy, visual, or motor deficits. To best represent the ED-user population, patients were approached by interviewers in both waiting rooms and treatment areas based on convenience. Eligible patients were identified by researchers with assistance from staff physicians and registered nurses to ensure they met inclusion criteria. After obtaining verbal patient consent, researchers verbally administered the questionnaire, using SurveyMonkey (San Mateo, CA) software to electronically record the anonymized responses. Data was collected throughout June–July 2018 at all three EDs during daytime hours. We obtained ethical approval from the University of Saskatchewan research ethics board prior to conducting the questionnaires.

The anonymous questionnaire (Appendix I) consisted of eight demographic questions, including age, gender, ethnicity, nationality, employment status, and whether the individual had a family doctor. If participants responded “yes” to “do you smoke cigarettes now?”, they were asked an additional 16 questions. These questions served to further evaluate participants’ smoking habits, nicotine dependence, readiness to quit, and receptiveness to potential ED-based smoking cessation interventions. To assess for correlations between smoking status and acuity level, chief complaint, and CTAS scores, these scores were inputted after the survey, but no other personal information was recorded. CTAS is a triaging tool used in EDs across Canada and internationally, which scores patients based on chief complaint, vitals, and other parameters.^20^ A scale of 1 (acute life-threatening condition) to 5 (non-urgent presentation) is used.

### Questionnaire Development

The questionnaire was developed for the purpose of this study and has not been validated previously. However, many individual survey questions have been validated in previous studies. Smoking status was assessed by asking “do you smoke cigarettes now?”, a phrase that has been validated previously, and correlated with breath carbon monoxide tests.^8^,^9^,^21^ Readiness to quit was determined by asking whether the patient wanted to quit, followed by whether he or she wanted to quit within the next one month or six months. Classifying when a patient wants to quit can predict their current stage of change. Previous literature in New Zealand has demonstrated that participants wanting to quit within a month are more likely to be in a preparation stage than participants who expressed just wanting to quit.^22^

We used the Fagerström Test for Nicotine Dependence (FTND) to stratify individuals’ nicotine addiction^23^ into categories of minimal, moderate, or high nicotine dependence. This tool is composed of six questions that explore the smoking characteristics of participants. The FTND has been validated as an accurate predictor of nicotine dependence internationally^24^,^25^ and is frequently used to determine nicotine dependence.^7^,^8^ Finally, participants who smoked were asked about willingness to participate in ED smoking cessation interventions including brief cessation counselling in the department, referral to quit smoking hotlines, and/or receiving a pamphlet about quitting. While not previously validated, phrasing of the brief cessation counselling question was identical to an Australian study on ED smoking prevalence.^7^ Based on previous literature, the majority of ED smoking cessation studies have also included referrals to smokers’ quit lines and pamphlets; thus, we included questions on patient receptiveness to those interventions.^15^

### Data Analysis

Data were analyzed using SPSS software (IBM Corp, Armonk, NY) to determine smoking prevalence and compared to Statistics Canada data using chi-square and Cochrane-Armitage trend tests. Statistics Canada is the federally legislated statistics office, which organizes national surveys and a census every five years. We determined the Saskatchewan smoking rate by dividing the number of people aged 18 years or older who smoked daily in Saskatchewan in 2016^2^ by 2016 census data of the Saskatchewan population aged 20 and older.^3^ Age grouping in these two parameters varied, which made it impossible to compare between 18 years and older populations. Instead we chose to use the number of individuals in the provincial population aged 20 years and older who reported smoking daily, as any error would over-represent the smoking prevalence. Lastly, we used chi-square tests to compare population differences between active smokers wanting to quit and receive ED therapy, and those opposed to quitting and receiving ED therapy.

## RESULTS

**Figure 1:**
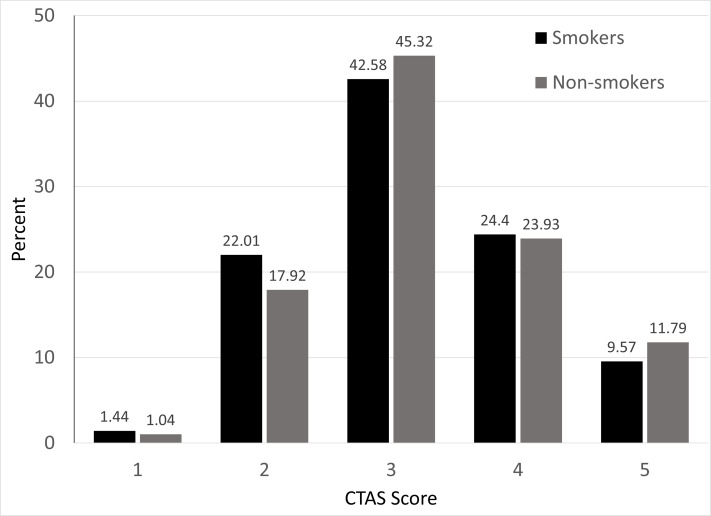
Comparison of Canadian Triage Acuity Scale scores between smoking and non-smoking ED patients. *CTAS*, Canadian Triage and Acuity Scale.

Of 1190 eligible participants who were approached to participate in the survey 112 declined, leaving a sample size of 1084 participants with 1078 disclosing smoking status. Of the completed questionnaires, 47.5% (n = 514) were completed at Royal University Hospital, 30.6% (n = 331) at St Paul’s Hospital, and 22.0% (n = 238) at Saskatoon City Hospital; these proportions are representative of the patient distribution between the three Saskatoon EDs. Across the three sites, 19.6% (n = 211) of ED patients self-reported current cigarette smoking (Table 1). There was no difference in prevalence rates among the three sites (*P* = 0.74). Using Statistics Canada data, we calculated that the prevalence of daily cigarette smokers in Saskatchewan in 2018 was 14.65%.^2^,^3^ Comparing to the prevalence of cigarette smoking in adult ED patients with the calculated Saskatchewan prevalence overall, the proportion of ED patients who currently smoke was significantly higher (95% confidence interval, 17.1–21.8%, *P* < 0.0001). No differences in CTAS score were noted between smoking and non-smoking cohorts, meaning that smoking patients on average did not present more acutely than non-smokers (Figure 1).

Variations between gender and citizenship status were minimal between groups. Interestingly, the 20–34 year age category made up a higher proportion of the smoking cohort (25.6%) compared to non-smokers (14.0%) (*P*<0.0001). Furthermore, 79.6% of ED patients who smoked indicated they had a family doctor, which was significantly lower than the non-smoking cohort at 91.4% (*P*<0.0001). The smoking cohort was also more likely to originate from countries outside of Canada, more likely to be currently employed or unemployed, and less likely to be retired or on long-term disability.

The majority of the smoking cohort (88.1%) indicated they had begun smoking before age 20 (Table 2). Additionally, 73.8% reported they were interested in quitting, while 37.1% reported that their current visit to the ED had caused them to consider quitting. Based on Fagerström scores, nearly half of the smoking cohort was classified as having a minimal nicotine dependence. Of the three suggested ED cessation interventions, participants were most receptive to receiving ED cessation counselling (62.4%), followed by receiving a smoking cessation pamphlet (56.2%), and lastly being referred to a smoker’s quit line (49.5%).

As demonstrated in Table 3, 51.4% (n = 211) of the smoking cohort indicated they were both interested in quitting and willing to receive ED-specific counselling. The demographics of smokers interested in quitting and those not interested were fairly homogenous. However, participants in the smoking cohort with less acute CTAS (ie, 4 or 5) scores were generally more receptive compared to those with more severe presentations. Incidentally, the smoking cohort at Royal University Hospital was significantly less receptive to quitting and receiving counselling in the ED than the other two urban hospitals (*P* = 0.006).

## DISCUSSION

Based on our findings, the prevalence of cigarette smoking among adult ED patients (19.6%) was significantly higher than the general population prevalence (14.65%).^2^,^3^ Similar to previous international studies assessing ED smoking habits, we also demonstrated an ED smoking prevalence that is significantly higher than the respective region.^5^–^8^ This increased ED smoking prevalence is likely a multifactorial result of government policy, socioeconomic status, and healthcare system structure. ED patients who smoke were found to be younger than the non-smoking cohort, which is consistent with previous studies.^6^–^8^ Additionally, ED patients who smoke also had lower rates of being retired or on long-term disability, and more likely to be either employed or unemployed. Similar employment trends have been previously demonstrated^7^ and is likely secondary to the younger age of the cohort. Lastly, a higher proportion of ED patients who smoked were originally from outside Canada, which could be attributable to differences in government tobacco regulation in their countries of origin.

Interest in quitting was also comparable to international studies: 73.8% of the ED smoking cohort expressed interested in quitting, which was similar to Australian and New Zealand studies of 69.7–74.9%.^7^,^8^ Furthermore, 51.4% (n = 108) of the smoking cohort were interested in both quitting and receiving ED-based support. Therefore, a targeted cessation intervention in the ED could potentially benefit a large number of active cigarette smokers. Identifying which patients within the smoking cohort might benefit from an intervention is difficult; however, CTAS 4 or 5 patients may be more interested in quitting than those with more acute CTAS scores. Quit attempts and prolonged abstinence rates have been demonstrated to be more efficacious in individuals with lower FTND scores.^26^ As the majority of ED patients who smoke were categorized as having minimal to moderate nicotine dependence (87.2%), ED counselling and interventions could prove beneficial for smoking abstinence.

All three proposed smoking cessation interventions have been previously trialed in other EDs. Cessation counselling while in the ED was considered the most favoured modality among patients; however, traditional counselling is limited by time constraints. The Ask-Advise-Refer motivational interviewing model is designed to take under three minutes,^27^ and has been previously validated in the ED through training emergency nurses to administer the intervention.^14^ Cessation pamphlets were also positively regarded by our ED patients, and could be used to connect them with community-based cessation resources. However, there is little evidence to support pamphlets as a stand-alone intervention.^15^ While ED patients were least receptive to receiving a referral to smoking cessation helpline, it could be a feasible method to follow up with patients. Multifaceted interventions with repeated patient interactions improve the likelihood of a successful quit attempt.^28^ However, not all ED cessation interventions have correlated to an improvement in patient abstinence rates.^15^ This suggests that implementing an ED smoking intervention could be effective for initiating cessation; however, pairing the intervention with community-based supports for follow-up would likely improve cessation rates.

Furthermore, participants within the New Zealand and Australian studies reported interest in quitting at 74.9% and 69.7%, respectively, which is similar to our calculated 73.8%.^7^,^8^ Patients’ willingness to undergo brief ED counselling (62.4%) was also comparable (60.3%).^7^ Similarly, receptiveness to receiving a smoking cessation pamphlet (56.2%) was comparable to interest in receiving a “quit smoking pack” in a New Zealand study (60.6%).^8^ With these similarities in patient receptiveness to ED cessation modalities between countries, it is possible that successful interventions trialed in one country may be generalizable to others. As 88.1% of our ED patients who currently smoke began smoking before the age of 20, it could prove beneficial to further explore the utility of pediatric EDs as a screening location for cigarette use.

Interestingly ED patients who smoked both in our study and in the New Zealand study were less likely than non-smokers to have a family doctor.^8^ While Canadian hospitals often have an inpatient-based smoking cessation protocol,^13^ this has limited utility for most ED patients who are discharged without admission. As smoking cessation support is traditionally in the scope of the primary care physician, some of our patients who smoke may not be getting cessation support elsewhere.^6^

We encourage our colleagues to assess smoking status in their respective EDs. This will help determine whether our results, and the results of previous studies, are generalizable to the rest of our respective countries. Our next steps will be to develop a smoking cessation intervention that will benefit patients while remaining feasible for the unique and fast-paced environment of the ED.

## LIMITATIONS

As we did not objectively measure whether a patient currently smoked, our data is based on self-reported responses, which could impact the validity of the study. Furthermore, our data was collected in 2018, while the most accurate available Statistics Canada data is from 2016, which could further impact validity. Additionally, our provincial prevalence calculation used the general population aged 20 years or older, while our data collected in the ED included people aged 18 years or older. It is possible this discrepancy could overestimate the provincial prevalence, making the ED smoking prevalence more significant. Due to the anonymity of the survey, it is possible that the questionnaire could have been completed more than once by the same participant; however, this is unlikely. As data collection was performed during daytime hours, it is also possible that patients presenting to the ED at night could have had a different smoking prevalence. Finally, the census questionnaire asked if patients smoked cigarettes “daily,” while we asked if they smoked cigarettes “now,” which could have created subjectivity of responses.

## CONCLUSIONS

The prevalence of cigarette smoking in Saskatoon adult ED patients was found to be higher than the respective provincial and national rates, which is consistent with literature from other comparable countries. Over 50% of actively smoking patients indicated they wanted to quit smoking and would be receptive to receiving cessation counselling in the ED. These findings prime the ED as a novel location for initiating a smoking cessation intervention that is feasible, despite the fast-paced environment and limited capacity to provide follow-up support.

## Supplementary Information



## Figures and Tables

**Table 1:** Characteristics of surveyed patients in three Saskatchewan emergency departments.

	Current smokers (n = 211, 19.6%)	Non-smokers (n = 867, 80.4%)	P-value
Age, years, n (%)			
18–19	2 (1.0)	3 (0.3)	<0.0001
20–34	54 (25.6)	120 (13.8)	
35–44	28 (13.3)	83 (9.6)	
45–54	43 (20.4)	106 (12.2)	
55–64	47 (22.3)	127 (14.7)	
≥65	37 (17.5)	428 (49.4)	
Gender, n (%)			
Male	114 (54.0)	429 (49.6)	0.248
Female	97 (46.0)	436 (50.4)	
Citizenship status, n (%)			
Canadian	202 (96.2)	828 (95.5)	0.325
Permanent resident	8 (3.8)	30 (3.5)	
Non-permanent resident	0	9 (1.0)	
Country of origin, n (%)			
Canada	197 (93.8)	753 (86.9)	0.005
Outside of Canada	13 (6.2)	114 (13.1)	
Family physician, n (%)			
Yes	168 (79.6)	792 (91.4)	<0.0001
No	43 (20.4)	75 (8.6)	
Employment status, n (%)			
Employed	91 (43.1)	307 (35.4)	<0.0001
Family caregiver	7 (3.3)	16 (1.9)	
Retired/long-term disability	71 (33.7)	480 (55.4)	
Student	5 (2.4)	16 (1.9)	
Unemployed	37 (17.5)	48 (5.5)	
Hospital, n (%)			
Royal University	104 (49.3)	408 (47.1)	0.736
St. Paul’s	65 (30.8)	265 (30.6)	
Saskatoon City	42 (19.9)	193 (22.3)	
CTAS, n (%)			
1	3 (1.4)	9 (1.04)	0.597
2	46 (22.0)	155 (17.9)	
3	89 (42.6)	392 (45.3)	
4	51 (24.4)	207 (23.4)	
5	20 (9.6)	102 (11.8)	

Among all patients surveyed, gender was missing for 2 patients, citizenship status was missing for 1 patient, country of origin was missing for 1 patient, hospital was missing for 1 patient, and CTAS scores were missing for 10 patients. Six patients did not disclose their smoking status. Among smokers, citizenship was missing for 1 patient, country of origin was missing for 1 patient, and CTAS scores were missing for 2 patients.

*CTAS*, Canadian Triage and Acuity Scale.

**Table 2:** Smoking-related characteristics of Saskatchewan emergency department patients who reported smoking (N = 211).

	n (%)
Age when smoking started	
18–19 years	185 (88.1)
20–34 years	21 (10.0)
35–44 years	2 (1.0)
45–54 years	2 (1.0)
Believe ED visit related to smoking	43 (20.5)
ED visit has caused quitting consideration	78 (37.1)
Interested in quitting smoking	155 (73.8)
If yes:	
Within 1 month	118/155 (76.1)
Within 6 months	149/155 (96.1)
Nicotine dependency (Fagerström score)	
7–10 (high)	27 (12.8)
4–6 (moderate)	79 (37.4)
<4 (minimal)	105 (49.8)
Would undergo ED cessation counselling, if available	131 (62.4)
Referral to smoker cessation helpline	104 (49.5)
Pamphlet provided	118 (56.2)

*ED*, emergency department.

**Table 3:** Smokers who expressed interest in quitting and were willing to receive cessation counselling in the emergency department.

	n (% wanting to quit)	P-value*
Age		
18–34 years	27 (49.1)	0.21
35–44 years	13 (46.4)	
45–54 years	25 (58.1)	
55–64 years	29 (61.7)	
≥ 65 years	14 (37.8)	
Age of initiation		
≤ 19 years	95 (51.4)	0.95
≥ 20 years	13 (52.0)	
Gender		
Male	54 (47.8)	0.25
Female	54 (55.7)	
Citizenship status		
Canadian	103 (51.2)	0.72
Permanent resident	5 (62.5)	
Country of origin		
Canada	102 (52.0)	0.68
Outside of Canada	6 (46.2)	
Family physician		
Yes	86 (51.5)	0.97
No	22 (51.2)	
Employment status		
Employed	46 (51.1)	0.69
Retired/Long-term disability	39 (54.9)	
Other	23 (46.9)	
Hospital		
Royal University	42 (40.4)	0.006
St. Paul’s	40 (61.5)	
Saskatoon City	26 (63.4)	
Fagerström score		
7–10 (high)	16 (59.3)	0.30
4–6 (moderate)	44 (55.7)	
<4 (minimal)	48 (46.2)	
CTAS Score		
1 or 2	19 (38.8)	0.16†
3	49 (55.1)	
4	27 (52.9)	
5	13 (65.0)	

Comparison of demographic data, nicotine dependence, and CTAS score of smoking cohort interested in quitting and receptive to cessation counselling, with the smoking cohort not interested in quitting. One current smoker was missing information for all above variables; one additional patient was missing citizenship status, one was missing country of origin, and one was missing CTAS.

* P-value by chi-square test/Fisher’s exact test;

† Cochran-Armitage trend test P = 0.06, suggesting that the proportion of good candidates for cessation counselling may increase with higher CTAS scores.

*CTAS*, Canadian Triage and Acuity Scale.
