# Supplementary material for: Prevalence of Cigarette Smoking Among Adult Emergency Department Patients in Canada
Source: West J Emerg Med. 2020 Nov 1;21(6):190–7. doi: 10.5811/westjem.2020.9.47731 (PMC7673889; doi:10.5811/westjem.2020.9.47731)
Supplement: Supplementary file 1 [file wjem-21-190-s001.docx]

**SUPPLEMENTAL FILES**

| **Appendix 1. Survey** | |
| --- | --- |
| Age: | 18-19, 20-34, 35-44, 45-54, 55-64, 65+ |
| Sex | Male, Female, Other |
| What ethnicity do you identify with the most? | *(text box)* |
| Citizenship Status | Canadian Citizen, Permanent Resident, Non-permanent resident |
| Country of Origin | *(text box)* |
| Do you have a family doctor? | Yes, No |
| What is your current employment status? | Employed, Student, Unemployed, Homemaker, Retired/Long term disability |
| Do you smoke cigarettes now? | Yes, No |
| **If Yes** | |
| How old were you when you started smoking? | <19, 20-34, 35-44, 45-54, 55-64, >65 |
| Do you think your visit to the emergency department is related to your smoking? | Yes, No |
| Has your visit to the emergency department today made you consider quitting? | Yes, No |
| Do you want to quit smoking? | Yes, No |
| Do you want to quit within the next month? | Yes, No |
| Do you want to quit within the next 6 months? | Yes, No |
| How many quit attempts (>24 hours) have you made in the last 12 months? | 0, 1, 2, 3+ |
| How soon after you wake up do you smoke your first cigarette? | - Within 5 minutes (3 points) - 6 to 30 minutes (2 points) - 31 to 60 minutes (1 point) - After 60 minutes (0 points) |
| Do you find it difficult to refrain from smoking in places where it is forbidden such as church, the library, or movie theatres? | - Yes (1 point) - No (0 points) |
| Which cigarette would you hate most to give up? | - The first one in the morning (1 point) - All others (0 points) |
| How many cigarettes do you smoke? (20 cigarettes are in a pack) | - 10 or less (0 points) - 11-20 (1 point) - 21-30 (2 points) - 31 or more (3 points) |
| Do you smoke more frequently during the first hours after waking than the rest of the day | - Yes (1 point) - No (0 points) |
| Do you smoke if you are so ill that you are in bed most of the day? | - Yes (1 point) - No (0 points) |
| Would you be willing to undergo a brief counselling cessation in the emergency department to assist you in quitting if this service were available? | Yes, No |
| Smokers quit line referral? | Yes, No |
| Pamphlet? | Yes, No |
| **CTAS and Chief Complaint then recorded for all patients** | |
